# Associations of nm23H1, VEGF-C, and VEGF-3 Receptor in Human Prostate Cancer

**DOI:** 10.3390/molecules19056851

**Published:** 2014-05-23

**Authors:** Zui-Su Yang, Yin-Feng Xu, Fang-Fang Huang, Guo-Fang Ding

**Affiliations:** Engineering Research Centers of Marine Organism Medical Products, Medical College of Zhejiang Ocean University, Zhoushan 316022, China

**Keywords:** human prostate cancer, VEGFR-3, VEGF-C, nm23H1

## Abstract

We studied the expression of the non-metastatic clone 23 type 1 (nm23H1) gene, vascular endothelial growth factor (VEGF)-C, and its receptor VEGFR-3 using an *in situ* hybridization technique and immunohistochemical analyses with prostate cancer tissues and adjacent benign tissues of 52 human archival cases. The association between VEGF-C expression, microlymphatic count (MLC), and staining intensity for nm23H1 and VEGFR-3 was used to evaluate tumor metastasis and survival rate. MLC values were significantly higher in tumorous tissue than in non-cancerous tissue. VEGF-C mRNA, VEGFR-3, and nm23H1 were highly expressed in tumorous tissue. VEGFR-3 expression was greater in VEGF-C mRNA-positive tumors than in VEGF-C mRNA-negative tumors. The association of VEGFR-3 expression with VEGF-C mRNA and MLC suggested that the poor prognosis and tumor metastasis associated with VEGFR-3 expression may be due, in part, to its role in promoting angiogenesis. VEGF-C expression was significantly associated with tumor lymphangiogenesis, angiogenesis, and immune response as a potent multifunctional stimulating factor in prostate cancer. Expression of nm23H1 was significantly inversely correlated with lymph node metastasis. Furthermore, there was a strong negative correlation between the expression of nm23H1, VEGF-C mRNA, and MLC. These findings provide important information for prophylactic, diagnostic, and therapeutic strategies for prostate cancer.

## 1. Introduction

Prostate cancer (PCa) is a prevalent, multifunctional, and heterogeneous disease, primarily affecting men over 50 years of age [1]. The incidence of PCa varies widely around the world, attributed to environmental, genetic, dietary, and hormonal factors, as well as their interactions [2,3,4,5,6]. Metastasis is the major cause of death from PCa, with the development of rich vascular networks of new lymphatic vessels and blood vessels in the tumor, namely angiogenesis, resulting in resistance to conventional therapies such as androgen ablation and cytotoxic chemotherapy [7]. A better understanding of the molecular mechanisms and genes involved in the initiation, progression, and metastasis of PCa, is necessary not only to help develop more effective therapeutic strategies, but also to advance basic cell science. It is important to elucidate the molecular mechanisms underlying the growth of new lymphatic vessels and the metastasis suppressor gene in human prostate tumors.

The molecules that induce lymphatic vessel development, including members of the vascular endothelial growth factor (VEGF) family and their receptors (VEGF-A, VEGF-B, VEGF-C, VEGF-D, placental growth factor, VEGFR-1, VEGFR-2, and VEGFR-3), are associated with the angiogenesis induced by most cancer-cell types and certain tumor stromal cells [8,9,10,11]. Jackson *et al.* [12] first showed a widespread distribution of VEGF in PCa specimens and suggested that the VEGF_165_ and VEGF_189_ isoforms, novel 90- and 110-kD forms that are detected in the specimens, contribute to the establishment or progression of PCa. Ferrer *et al.* [13], Balbay *et al.* [14], Duque *et al.* [15], Strohmeyer *et al.* [16], and Mazzucchelli *et al.* [17] also reported increased levels of VEGF in PCa based on immunohistochemical findings. In addition, Krupski *et al.* [18], Jackson *et al.* [19], and Puyromaure *et al.* [20] studied the role of VEGF in the tissue-specific *in vivo* growth of PCa cells to examine its biologic impact on prostate tumors to promote angiogenesis and autocrine regulation of tumor growth, finding that VEGF acts as a multifunctional cytokine in prostate tumors and may have a prognostic impact in clinically-localized PCa.

Furthermore, among the VEGF isoforms, Rinaldo *et al.* [21] reported an increase in VEGF-C expression in human PCa cells after androgen withdrawal. In general, VEGF-C interacts with VEGFR-2 to stimulate angiogenesis [22]. Another important lymphangiogenic factor, VEGFR-3, present in the endothelial cells of tumor blood vessels [23], promotes the metastasis of cancer cells via the lymphatic system [24]. In contrast, metastasis-suppressor genes in PCa, such as the nonmetastatic clone 23, maspin, HP1^Hsα^, and gelsolin genes, suppress the formation of spontaneous overt metastases [25,26,27,28]. Kauffman *et al.* [26] identified seven genes that suppress metastasis without affecting primary tumor growth: KAI1, CD44, mitogen activated protein kinase 4, nonmetastatic clone 23 type 1 (nm23H1), nm23H2, KiSS1, and BrMS1. Three of these genes (KAI1, CD44, and mitogen activated protein kinase 4) act as metastasis suppressor genes of PCa, while the others have yet to be tested in this cancer type.

The nm23H1 gene is a putative tumor metastasis suppressor that might be associated with the expression of VEGF-C and its receptor. In the present study, we studied the expression of the nm23H1 gene, and VEGF-C and its receptor VEGFR-3 in PCa using an *in situ* hybridization technique. Immunohistochemical analyses using antibodies against nm23H1 and VEGFR-3 were also performed in PCa and adjacent benign tissues of 42 human archival cases in China. Furthermore, we examined the expression of VEGF-C mRNA, microlymphatic count (MLC), and the intensity of staining for nm23H1 and VEGFR-3 to evaluate tumor metastasis and the 5-year survival rate. Our aim was to reveal the expression of nm23H1, VEGF-C and its receptor VEGFR-3, and their association with PCa metastasis to elucidate the functional significance and mechanisms of nm23H1 and VEGF-C in PCa. These findings provide original data of Chinese PCa patients and important information for prophylactic, diagnostic, and therapeutic strategies for PCa.

## 2. Results and Discussion

### 2.1. Results

**Figure 1:**
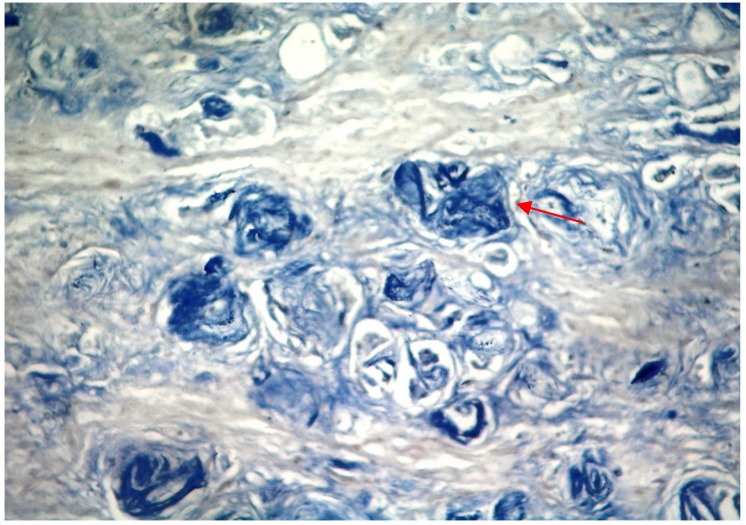
Photomicrograph showing strong VEGF-C mRNA expression in cells of prostate cancer tissue, with positively-stained granules in the cytoplasm and nucleolus of tumor cells (*in situ* hybridization, magnification 400×).

**Figure 2:**
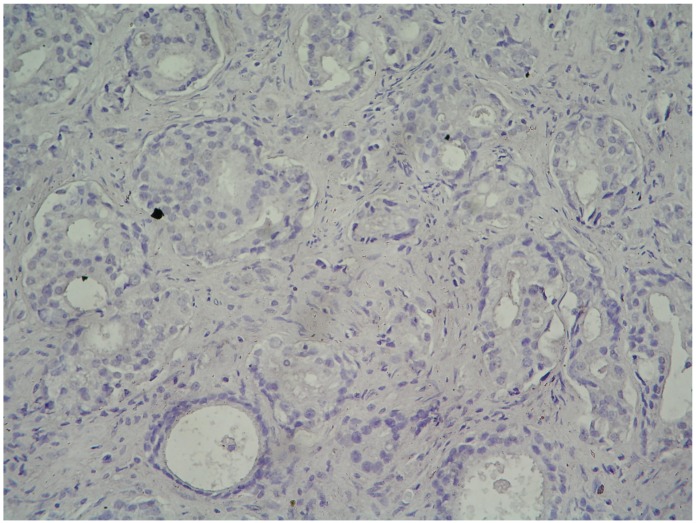
Photomicrograph showing negative staining of VEGF-C mRNA in cells of adjacent benign tissue (*in situ* hybridization, magnification 400×).

VEGF-C hybridization was considered positive when the ratio of cancer cells whose cytoplasm was stained blue or purple black was greater than 10% (Figure 1). The adjacent benign tissue was not stained (Figure 2). Among the 42 specimens, VEGF-C mRNA expression was detected in 19 tumor specimens with a positive ratio of 45%, from which 7 patients were in TNM stages I and II, and 12 patients were in TNM stages III and IV (Table 1). In addition, VEGF-C mRNA expression was detected in 26% (8/31) of those negative for lymph node metastasis and 100% (11/11) in those positive for lymph node metastasis (*p* < 0.05; Table 2).

**Figure 3:**
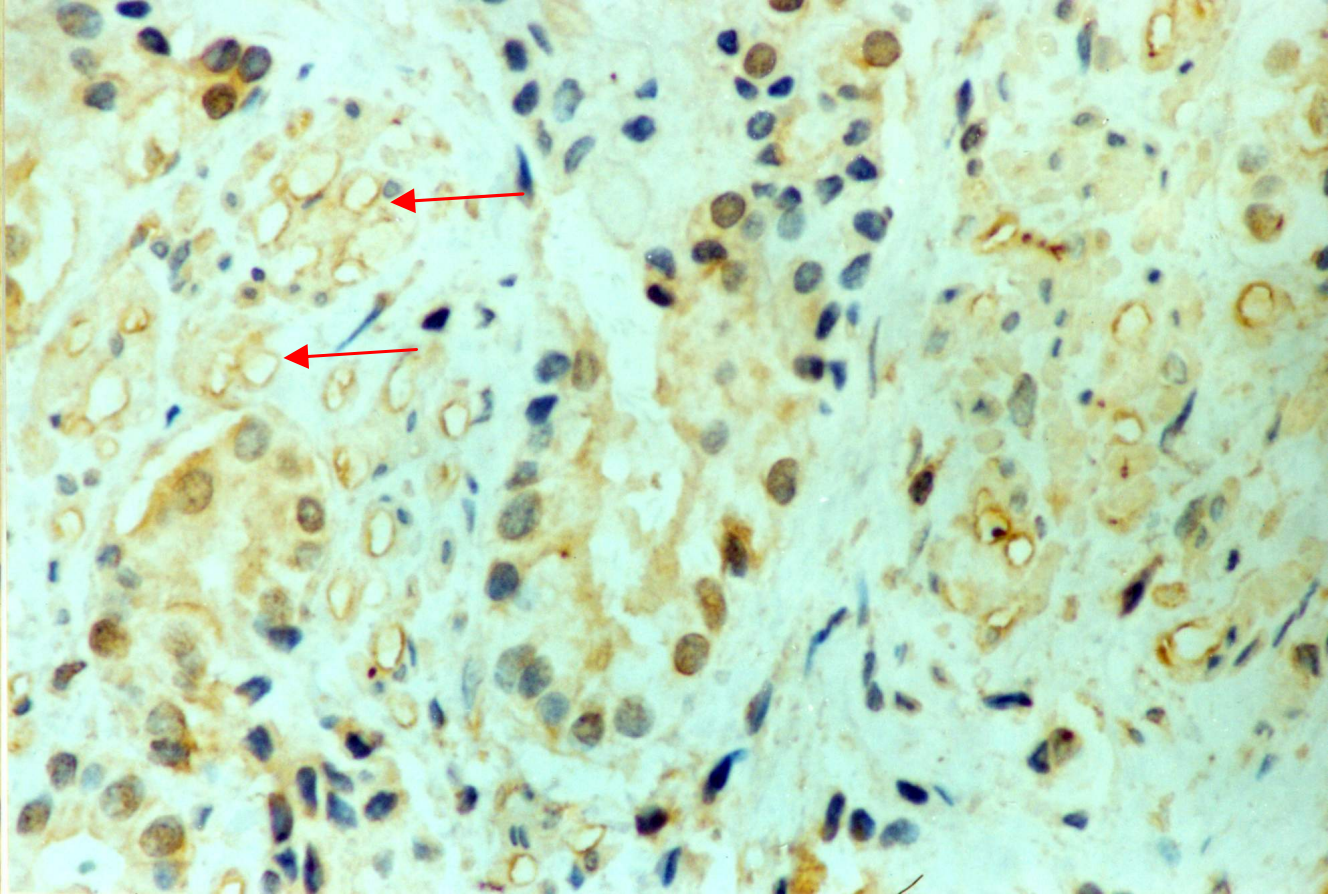
Photomicrograph showing immunohistochemical staining of VEGFR-3 in cells of prostate cancer tissue. Arrows indicate high intratumoral microlymphatic density (EnVisionTM, magnification 400×).

VEGFR-3 was expressed mainly in the endothelial cells of lymphatic vessels, as indicated by brown or brown-yellow staining (Figure 3). VEGFR-3 positive lymphatic vessels were observed mainly in the stroma between PCa tissues. The microvessel walls had an irregular morphology and no new lymphatic vessels were observed in the center of the PCa tumor. MLC values were greater in PCa tissues (mean ± standard error, 8.61 ± 2.67/mm^2^) than in the adjacent benign tissues (4.51 ± 2.64/mm^2^; Table 2). The mean MLC values were 11.16 ± 1.39/mm^2^ for specimens positive for VEGF-C mRNA and 6.93 ± 1.80/mm^2^ for specimens negative VEGF-C mRNA. In addition, MLC values were higher in in stage III and IV PCa tissues or in patients with lymph node metastases than in stage I and II tumors or in patients without lymph node metastases (*p* < 0.05), or in the adjacent benign tissues (*p* < 0.01). The MLC of stage I and II PCa tissues was not significantly different than the MLC of the adjacent benign tissue. Consequently, there was no statistically significant relationship regarding the difference in the expression of VEGF-C mRNA and MLC in PCa tissues of different histopathologic grades.

**Table 1:** Clinical characteristics of 42 PCa patients.

Characteristics	Cases (%)
*Age (years)*	
<65	16 (38.10%)
≥65	26 (61.90%)
*Lymph node metastasis*	
N0	
N1a	31 (73.81%)
N1b	11 (26.19%)
*TNM clinical stage*	
Stage I	12 (28.57%)
Stage II	10 (23.81%)
Stage III	9 (21.43%)
Stage IV	11 (26.19%)
*Gleason score*	
≤4	7 (16.67%)
5–6	9 (21.42%)
7	19 (45.24%)
≥8	7 (16.67%)
*Pathologic grade*	
Grade I	12 (28.57%)
Grade II	9 (21.43%)
Grade III	11 (26.19%)
Grade IV	10 (23.81%)

**Table 2:** Relationship between nm23H1, VEGF-C, MLC, and clinicopathology of PCa tissue.

Clinicopathological	No. of cases	nm23H1-positive cases (%)	VEGF-C-positive cases (%)	MLC /mm^2^ mean ± dev
**adjacent benign tissues**				4.51 ± 2.64
*Pathologic grade*				
I and II	21	6 (28.27)	9 (42.86)	8.61 ± 2.67
III and IV	21	13 (61.91)	10 (47.62)	7.92 ± 2.04
*TNM stage*				
I and II	22	20 (90.91)	7 (31.82)	7.45 ± 2.91
III and IV	20	9 (45.00) **	12 (60.00) *	9.79 ± 2.68 *
*Lymph node metastasis*				
Negative	31	25 (80.65)	8 (25.81)	6.93 ± 1.80
Positive	11	4 (36.36) **	11 (100) **	11.16 ± 1.39 *

* *P* < 0.05, ** *P* < 0.01.

**Figure 4:**
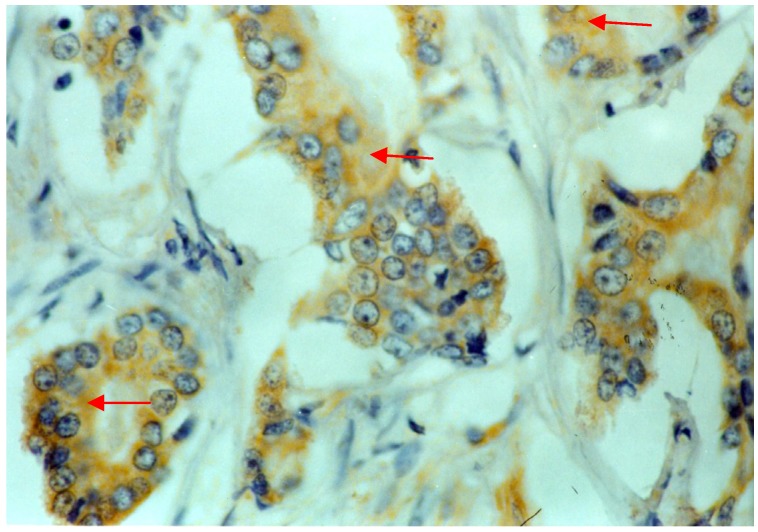
Photomicrograph showing nm23H1 expression in cells of prostate cancer tissue, with positive brown-yellow granules in the cytoplasm (EnVisionTM, magnification 400×).

Figure 4 shows a representative microphotograph of nm23H1 expression with brown-stained granules mainly located in the cytolymph, based on immunohistochemistry in PCa cells. In the present study, 1 adjacent benign tissue specimen and 29 PCa tissue specimens were positive for nm23H1 expression, with ratios of 10% and 69%, respectively. The nm23H1 staining intensity in PCa tissues was significantly greater compared to that of the adjacent benign tissue (*p* < 0.01). Furthermore, nm23H1 expression levels in the tumor varied among the TNM stages (*p* < 0.05). The ratio of positive expression was 91% (20/22) in stage I and II tumors, and 45% (9/20) in stage III and IV tumors (*p* < 0.05). In addition, nm23H1 expression was positive in 81% (25/31) of patients with negative lymph node metastasis and in 36% (4/11) of patients with positive lymph node metastasis (*p* < 0.05, Table 2).

Of the 42 PCa patients, 13 died within 5 years with a 5-year disease-specific mortality rate of 31%. The correlation between the expression of nm23H1, VEGF-C mRNA, and the MLC with the survival rate of sampled PCa patients after prostatectomy is shown in Table 3. Survival was significantly higher in PCa patients that were moderately or strongly nm23H1-positive, or had high MLC, compared with those who were weakly nm23H1 positive or nm23H1 negative, or had low MLC (*p* < 0.05). VEGF-C mRNA was inversely related to survival in the sampled PCa patients. 

**Table 3:** Relationship between nm23H1, VEGF-C mRNA expression, and MLC, and survival rate in PCa patients.

	No. of cases	5-year survival ratecase (%)	5-year death rate case (%)
	42	29	13
*MLC*		5.73 ± 2. 09 mm^2^	10.82 ± 2.51 mm^2^
*nm23H1*			
− and +	19	9 (47.37)	10 (52.63)
++ and +++	23	20 (86.96）	3 (13.04)
*VEGF-CmRNA*			
negative	23	22 (95.65)	1 (4.35)
positive	19	7 (36.84)	12 (63.16)
*Pathologic grade*			
I and II	21	17 (80.95)	4 (19.05)
III and IV	21	12 (57.14)	9 (42.86)

### 2.2. Discussion

An understanding of the molecular mechanisms underlying the association between VEGF-C expression and its receptor and genes involved in PCa is crucial toward elucidating the factors involved in PCa initiation, progression, metastasis, and potential therapeutic strategies. VEGF-C and one of its receptors, VEGFR-3, are associated with lymphatic metastasis mainly via tumor lymphangiogenesis in both animal models and human tumors [29,30,31]. Numerous studies have revealed increased VEGF-C expression in PCa. In the present study, we therefore focused on VEGFR-3, nm23H1, lymphatic vessel counts, and hyperplasia, metastasis and survival rate in human PCa tissues to investigate their potential roles in tumor cell proliferation and PCa metastases using *in situ* hybridization and immunohistochemistry techniques.

The present study of 42 PCa patients demonstrated that positive expression of VEGF-C mRNA was associated with TNM stage and lymph node metastasis of PCa tumors. Expression of VEGF-C mRNA in cancer cells was observed in 45% of prostate tumors, of which 32% were in TNM stages I and II, and 60% were in TNM stages III and IV. Simultaneously, positive expression of VEGFR-3 was detected mainly in the endothelial cells of lymphatic vessels. The MLC was higher in PCa tissues than in the adjacent benign tissues. Furthermore, MLC was also higher in tissues positive for VEGF-C mRNA than in those negative for VEGF-C mRNA. VEGF-C expression was positively correlated with VEGFR-3 expression in PCa cells. Our results indicate that a concomitant high expression of both VEGF-C and VEGFR-3 in cancer cells is associated with lymph node metastasis, suggesting a significant role of VEGF-C/VEGFR-3 in the proliferation of tumor lymphatic vessels and lymph node metastasis of PCa tumors. In fact, many tumors and ectogenetic tumor cells excrete VEGF-C to accelerate tumor growth and proliferation [32]. VEGF-C integrates and activates VEGFR-2 in blood vessel endothelial cells to induce new blood vessel formation, as well as VEGFR-3 in lymphatic vessel endothelial cells to induce new lymphatic vessel formation. Valtola *et al.* [33] reported that the VEGF-C/VEGFR-3 signaling pathway is associated with the formation and growth of blood vessels and lymphatic vessels. VEGF-C stimulates karyokinesis and proliferation of lymphatic vessel endothelial cells, and accelerates the growth and metastasis of lymphatic vessels by MEK/ERK and P13 kinase/Akt pathways. In the present study, we confirmed that tumor cells synthesize and excrete VEGF to accelerate the growth of lymphatic vessels in the tumor tissue. Due to the incomplete basement membrane in new lymphatic vessels and the large interstices among endothelial cells, tumor cells can easily invade and metastasize via the lymph system.

Tumor metastasis is a complex and dynamic process. The nm23H1 gene, a tumor metastasis suppressor, is located at human chromosome 17q21.3. Steeg *et al.* [34] first isolated the nm23H1 gene as a metastasis suppressor gene by differential screening of a cDNA library from low and high metastatic murine melanoma cell lines. Garinis *et al.* [35] demonstrated that the nm23H1 gene plays an important role in suppressing metastasis in sporadic colorectal carcinomas. The nm23H1 gene and its expression of nucleoside diphosphate kinase A suppress tumor metastasis by their involvement in cell signal transduction and microtubule assembly. In the present study, higher levels of nm23H1 gene expression were detected in PCa patients in TNM stages I and II than in those in TNM stages III and IV. We confirmed that the nm23H1 gene suppressed the metastasis of PCa tumors. In addition, weak, or even absent nm23H1 gene expression in PCa stage IV was observed during the growth and proliferation of PCa tumors, inducing high MLC levels and strong tumor metastasis. The biologic behavior of invasive PCa cells led to low survival rate and bad prognosis. In contrast, when the nm23H1 gene was strongly expressed, the MLC level was low and the tumors showed little metastasis, and those patients had a higher survival rate and better prognosis. Decreasing positive expression of nm23H1, increasing positive expression of VEGF-C mRNA, and high MLC are all signs of poor prognosis in PCa patients. The expression of nm23H1 and VEGF-C in PCa tissues was strongly negatively associated with their expression in the adjacent benign tissue. In addition, VEGF-C, nm23H1, and VEGFR-3 staining are strong predictors of overall survival. Geutz *et al.* [36] also reported that angiogenesis, assessed by MVD or VEGF expression, is significantly inversely related to survival in patients with colorectal cancer and breast cancer. Therefore, these experimental data contribute to an accurate prediction of the clinical progress of therapy and the prognosis in PCa patients, and allow us to estimate metastasis, prognosis, and survival from PCa.

## 3. Experimental

### 3.1. Tissue Samples

In the present study, PCa tissues samples were obtained between 1997 and 2000 from 42 patients with ages ranging from 51 to 84 years old (median age: 72 years) in Zhoushan People’s Hospital and Zhejiang People’s Hospital (Zhejiang. China), as shown in Table 1. The patients had undergone prostatectomy without preoperative hormonal therapy, chemotherapy and actinotherapy. All the sampled patients were clinically classified into stage I (12 patients), stage II (10 patients), stage III (nine patients) and stage IV (11 patients) according to the international TNM classification system of UICC, and categorized into Grade I (12 patients, well differentiated), Grade II (nine patients, moderately differentiated), Grade III (11 patients, poorly differentiated) and Grade IV (10 patients, non-differentiated) according to the World Health Organization grading system. In addition, the Gleason score of the 42 specimens was 2–4 in seven patients, 5–6 in nine patients, 7 in 19 patients and 8–10 in seven patients. Furthermore, 10 cases of adjacent nontumorous tissue specimens were tested for comparison in the study. All the specimens were cut into 4 µm thick, formalin-fixed, dehydrated, paraffin-embedded sections for EnVision^TM^ immunohistochemistry (IHC), and into 10 µm thick for *in situ* hybridization. 

### 3.2. In Situ Hybridization

The paraffin-embedded sections were dewaxed with dimethylbenzene, rehydrated by gradient EtOH/H_2_O, treated with 0.3% H_2_O_2_/MeOH for 30 min, dipped into 0.2 mL HCl for 20 min, and washed by DEPC water. Then the sections were added 50 μg/mL protein enzyme K, incubated at 37 °C for 30 min, dipped into the 0.2% glycin-PBS buffer for 10 min and washed by PBS buffer. After fixed by 4% polyformaldehyde-PBS liquor, washed by PBS buffer and dipped in 0.25% acetic anhydride for 10 min, each section was added 20 μL hybridized liquor with 2.5 mg/L VEGF-C probe, incubated at 42 °C for 16 h, balanced in 2 × SSC (contain 50% methanamide), followed by 2 × SSC, 0.5 × SSC, 0.1 × SSC for 15 min twice. The specific oligonucleotide probes labeled with digoxin on the 5'-end were made of two sequences: 5'-TGTACAAGTGTCAGCTAAGG-3' and 5'-CCACATCTATACACACCTCC-3' (Dingguo Biotechnology, Beijing, China). The sections were washed with PBS buffer twice and then checked with digoxin reagent box. Subsequently, the sections were added the retarder at room temperature for 30 min, rabbit-anti-digoxin-BSA liquor at room temperature for 1h, washed by PBS for 5 min 4 times, and then added alkalescent phosphate enzyme-sheep-anti-rabbit IgG liquor, incubated at 37 °C for 1h, washed by PBS for 5 min 4 times. After that, it was color-produced by chromogenic reagent at 4 °C in icebox to be examined every 15 min. The sections were then rinsed in distilled water at room temperature, counterstained by nucleus fixed red reagent, washed again by water, dehydrated, vitrified by dimethylbenzene, mounted by neuter balata. The negative comparison was hybridized with no-probe liquor.

### 3.3. Immunohistochemistry

Immunohistochemical staining was performed on the sections with the thickness of 4 µm. The paraffin-embedded sections were first dewaxed, rehydrated, treated by microwave, and then placed in 3% H_2_O_2_ for 20 min to quench endogenous peroxidse activity. After blocked with 10% normal sheep serum for 30 min, the sections were first incubated with anti nm23H1 antibody (GA009601, DAKO, Carpinteria, CA, USA) and anti-human VEGFR-3 antibody (rabbit polyclonal, 1:100 diluted, SC-321, Santa Cruz Biotechnology, Santa Cruz, CA, USA) overnight at 4 °C, and then incubated in the Chem MateTM Dako EnVision^TM^ Detection kit (DakoCytomation A/S, Glostrup, Denmark) at room temperature for 1h. The sections were washed off with 0.01mol/L PBS (3 × 5 min) between the steps. Finally, the sections were stained with DAB-H_2_O_2_, counterstained with hematoxylin, dehydrated and mounted with neuter balata. Sections incubated in PBS buffer and nonimmune serum instead of anti nm23H1 antibody s were used for the negative control.

### 3.4. Microscopic Assessment of VEGF-C mRNA, VEFGR-3, nm23H1 Expressions and MLC

A semiquantitative subjective method was carried out to determine nm23H1, VEGF-C and VEGFR-3 expressions in PCa tissues and adjacent nontumorous tissues. The expression of nm23H1 was positive when immunostained as brown granule mainly located in the nuclei and cytolymph. Semiquantitative scoring was carried out for the microscopic assessment of the intensity of immunostaining and the ratio of the positive cells with the expression of nm23H1 using a 4-scale system: − = negative (0%); + = weak-positive (1%–25%); ++ = moderate-positive (26%–50%) and +++ = strong-positive (51%–75%), observed with an Olympus BX-41 microscope. Simultaneously the VEGF-C hybridization was positive when the ratio was more than 10% of the cancer cells whose cytoplasm were marked in blue or purple black [37]. VEGFR-3 vessels were assessed under 100 magnification in a field area of 2.0 mm^2^ in five areas. MLC assessment was determined on the sections with VEGFR-3 IHC staining under 200 magnification in a grid area of 0.16 mm^2^ with a Leica Qwin computer image analysis system, using the criteria of Weidner [38]. Five areas of high vascular density (hotspots) were selected and counted on each section, and MLC was determined.

### 3.5. Statistical Analysis

The chi-square test with Yates correction was used to evaluate the scale system of the expression of nm23H1 and VEGF-C mRNA in cancer cells. Statistical analyses were performed using SPSS for Windows (SPSS Inc., Chicago, IL, USA) for the assessment of MLC. Threshold for significance was statistically *p* < 0.05. Student’s *t*-test was used to compare the mean and median values of between high and low VEGF levels.

## 4. Conclusions

In conclusion, the findings of the present study confirmed that the nm23H1 gene suppresses hyperplasia and metastasis of PCa, thereby improving the survival rate of PCa patients, while VEGF-C mRNA accelerates hyperplasia of the lymphatic vessels induced by the tumor and plays an important role in lymph node metastasis. Expression of VEGFR-3 was highly correlated with tumor metastasis. The high increase of MLC in PCa tissue indicates the hyperplasia of new lymphatic vessels and may be a biologic marker for tumor metastasis and survival. Furthermore, nm23H1 expression was strongly negatively associated with VEGF-C expression. Further investigation with larger groups of PCa patients is required to clarify the reliability of VEGF-C expression and its receptor VEGFR-2 as indicators of new blood vessel formation to predict metastasis, prognosis, and survival of human PCa patients.
